# A numerical study on the effect of CO_2_ addition for methane explosion reaction kinetics in confined space

**DOI:** 10.1038/s41598-021-99698-8

**Published:** 2021-10-20

**Authors:** Jingyan Wang, Yuntao Liang, Fuchao Tian, Chengfeng Chen

**Affiliations:** 1 China Coal Research Institute, Beijing, 100020 China; 2 State Key Laboratory of Coal Mine Safety Technology, China Coal Technology and Engineering Group Shenyang Research Institute, Fushun, 113122 China; 3 College of Emergency Management and Safety Engineering, China University of Mining and Technology (Beijing), Beijing, 100080 China

**Keywords:** Energy science and technology, Engineering

## Abstract

To explore the influence of the CO_2_ volume fraction on methane explosion in confined space over wide equivalent ratios, the explosion temperature, the explosion pressure, the concentration of the important free radicals, and the concentration of the catastrophic gas generated after the explosion in confined space were studied. Meanwhile, the elementary reaction steps dominating the gas explosion were identified through the sensitivity analysis. With the increase of the CO_2_ volume fraction, the explosion time prolongs, and the explosion pressure and temperature decrease monotonously. Moreover, the concentrations of the investigated free radicals also decrease as the increase of the CO_2_ volume fraction. For the catastrophic gas, the concentration of the gas product CO increases and the concentrations of CO_2_, NO, and NO_2_ decrease as the volume fraction of CO_2_ increases. When 7% methane is added with 10% CO_2_, the increase rate of CO is 76%, and the decrease rates of CO_2_, NO, and NO_2_ are 27%, 37%, and 39%, respectively. If the volume fraction of CO_2_ is constant, the larger the volume fraction of methane in the blend gas, the greater the mole fraction of radical H and the lower the mole fraction of radical O. For radical OH, its mole fraction first increases, and then decreases with the location of peak value of 9.5%, while the CO concentration increases with the increase of the methane concentration. For all the investigated volume fraction of methane, the addition of CO_2_ reduces the sensitivity coefficients of each key elementary reaction step, and the sensitivity coefficient of reaction promoting methane consumption decreases faster than that of the reaction inhibit methane consumption, which indicates that the addition of CO_2_ effectively suppresses the methane explosion.

## Introduction

Mine gas explosion accidents are one of the biggest factors, which endangers the safe production in coal mines. These accidents cause serious economic losses and casualties^1^. In recent years, with the continuous increase in coal production, the gas explosion accidents have occurred frequently^2,3^.

To prevent the occurrence of gas explosions, many relevant researches had been conducted in the field of inert gas explosion suppression. In terms of the explosion suppression experiments, Lu et al. designed a device that can automatically eject nitrogen during the explosion process. The effects of injection pressure, injection timing, and nozzle arrangement on the explosion suppression function were studies. The results showed that successful explosion suppression can be achieved when the nitrogen pressure reaches or exceeds 0.3 MPa^4,5^. Cao et al. studied the suppression effect of ultrafine mist on methane/air explosions. With the increase of ultrafine water/NaCl solution mist, the flame propagation speed, the maximum explosion overpressure, and the maximum pressure rising rate descended^6–8^. Based on the eddy dissipation concept combustion model, Wang et al. studied the mechanism and effect of ultrasonic water mist on suppressing gas explosion through experiments and EDC(Eddy-Dissipation Concept) combustion model^9^. Liang et al. investigated the influence of the nitrogen fraction in the blend of on the unstretched laminar flame propagation velocity, unstretched laminar combustion velocity, Markstein length, flame stability, and maximum combustion pressure. It was found that above parameters decrease distinctly with the increase of nitrogen fraction in the gas mixture^10^. Qian et al. obtained a fitting formula through experiments under different conditions, which can predict the explosion limit of methane at any ratio of N_2_ to CO_2_. They reported that the limit oxygen volume fraction decreases linearly with the increase in N_2_ content in the mixture^11^. Furthermore, some researches had been carried out to research the inhibition effect of N_2_, CO_2_ and N_2_/CO_2_ mixture on gas explosion, it was found that both N_2_ and CO_2_ can inhibit the gas explosion, and the inhibition effect on high concentration gas is better. At the same time, the higher the volume fraction of CO_2_ in the mixed gas, the better the inhibition effect^12–14^. The above researches show that the inert gas can inhibit the explosion, to deeply understand the behavior, many simulation works are performed.

Luo et al. used the (DFT) B3LYP/6-31G methods of density functional theory and the GRI-Mech 3.0 to analyze the related elementary reactions. The results indicated that the NH_3_ could achieve explosion suppression by competing the free radicals H and OH, and the reactant of O_2_ with CH_4_^15,16^. Liang et al. and Wang et al. found that the increase of the water content in the mixed gas can promote the generation of CO_2_ but reduce the intensity of the gas explosion, and inhibits the generation of harmful gases, such as CO, NO, and NO_2_^17,18^.

Lu et al. suggested that the H_2_O acts as the third body in the explosion process, which directly participated in the ternary collision reaction existing in the form of inert molecules. It would collide with the free radicals and the free atoms to destroy the chain carrier, which reduces the concentration of active centers in the chain reaction, and achieve the explosion suppression^19^. Ren et al. modified the reaction mechanism of GRI-Mech 3.0 by assuming that the N_2_, CO_2_, and H_2_O only participated in the inhibition process as the third body. The physical and chemical effects of the three inert gases on the laminar combustion velocity, adiabatic flame temperature, and net heat release rate under different methane equivalence ratios(Ф = 0.8, 1.0 and 1.2)were analyzed^20^. Jia et al. indicated that the N_2_, CO_2_, and H_2_O reduced the sensitivity of the elementary reaction steps dominating the gas explosions and the inhibition effect of CO_2_ and H_2_O were better than that of the N_2_^1,2,21^. Li et al. pointed out that the addition of N_2_, CO_2_, and H_2_O would strongly inhibit the generation of free radicals CH_3_ and HCO. The inhibitory effect of CO_2_ and H_2_O is not only from their participation in the three-body collision reaction, but also from their participation in another chain reactions^22,23^.

Though a number of experiments and simulation had been performed to investigate the suppression effect of inert gas on methane explosion, most of the previous studies focused only on the independent influences of different volume fractions of inert gas on methane explosion mechanism under stoichiometric ratio condition. Because the working condition of coal mine is complicated, and the inhibition effect may be different in different conditions. However, the influence of inert gases with different volume fractions on explosions over wide methane equivalence ratios has not been reported. In this study, the influence of CO_2_ volume fraction on methane explosion in confined space under different methane equivalent ratios was investigated to provide a theoretical basis for the improvement of the inert gas explosion suppression mechanism under complex working conditions.

## Mathematical model

### Governing equation

The composition equation is as follows.

1 $$\frac{{dY_{i} }}{dt} = v\mathop w\limits^{ \bullet }_{i} M_{i} \left( {i = 1,2, \ldots ,k_{g} } \right)$$

2 $$\mathop w\limits^{ \bullet }_{i} = \sum\limits_{k = 1}^{{N_{g} }} {v_{ik} } K_{fk} \prod\limits_{j = 1}^{{k_{g} }} {\left[ {X_{j} } \right]}^{{V_{ik}^{^{\prime}} }} \left( {j = 1,2, \ldots ,k_{g} } \right)$$

3 $$K_{fk} = A_{k} T^{{b_{k} }} \exp \left[ {\frac{{ - E_{k} }}{RT}} \right]\left( {k = 1,2, \ldots ,N_{g} } \right)$$

where *Y*_*i*_, *w*_*i*_, and *M*_*i*_ denote the mass fraction, chemical reaction rate, and molecular weight of the substance *i*, respectively, *t* is the time, *v*, *R*, and *T* represent the specific heat capacity, gas constant, and temperature of the mixture, respectively, and *N*_*g*_ and *k*_*g*_ are the total number of reaction steps and groups, respectively. The total number of points is the reverse stoichiometric coefficient, forward stoichiometric coefficient, and the difference between the forward and reverse stoichiometric coefficients of substance *i* in elementary reaction *k*. Here, *K*_*fk*_ is the rate constant of the positive reaction in the elementary reaction *j*, [*X*_*j*_] is the molar concentration of component *j*, and *A*_*k*_, *b*_*k*_, and *E*_*ak*_ are the pre-exponential factors, temperature index, and reaction activation energy of the elementary reaction *k*, respectively.

The energy equation is

4 $$c_{v} \frac{dT}{{dt}} + V\sum\limits_{i = 1}^{{k_{g} }} {e_{i} } \mathop w\limits^{ \bullet }_{i} M_{i} = 0$$

where *c*_*v*_ is the constant volume specific heat of the mixed gas, and *e*_*i*_ is the internal energy of component *i*.

### Sensitivity analysis

Sensitivity analysis is a method to determine the sensitivity factors that have an important impact on the overall response from multiple uncertain factors^24^.

Assuming a variable, it is expressed as

5 $$\frac{dZ}{{dt}} = F\left( {Z,t,a} \right)$$

where *Z* = (*Y*_*1*_*,Y*_*2*_*…,,*$Y_{{k_{g} }}$)^*t*^ is the mass fraction of each component, and *a* = *(A*_*1*_*,A*_*2*_*,…*$A_{{N_{g} }}$*)* is the prefactor of each elementary reaction.

6 $$w_{l,i} = \frac{{\partial Z_{l} }}{{\partial a_{i} }}$$

where *w*_*l,i*_ is the sensitivity coefficient, *Z*_*l*_ is the variable number *l*, and *a*_*i*_ is the prereference factor of the reactions *i*.

As the derivation of Eq. (6), one obtains

7 $$\frac{{dw_{l,i} }}{dt} = \frac{{\partial F_{l} }}{\partial Z}w_{l,i} + \frac{{\partial F_{l} }}{{\partial a_{i} }}$$

### Reaction mechanism

The total chemical reaction formula of gas explosion is CH_4_ + 2(O_2_ + 3.76N_2_) → CO_2_ + 2H_2_O + 7.52 N_2_ + 882.6 kJ/mol, GRI-Mech 3.0 is selected as the chemical reaction mechanism of methane combustion, the mechanism includes 53 species and 325 elementary reactions^25^. The study is performed by using a closed homogeneous 0-D reactor in CHEMKIN-Pro. Table 1 shows some key elementary reaction steps in the detailed mechanism of gas explosion chain reaction.

**Table 1:** Main reactions affecting the change of free radicals.

Reaction step	Elementary reaction
R32	O_2_ + CH_2_O < = > HO_2_ + HCO
R38	H + O_2_ < = > O + OH
R52	H + CH_3_(+ M) < = > CH_4_(+ M)
R53	H + CH_4_ < = > CH_3_ + H_2_
R57	H + CH_2_O(+ M) < = > CH_3_O(+ M)
R98	OH + CH_4_ < = > CH_3_ + H_2_O
R118	HO_2_ + CH_3_ < = > O_2_ + CH_4_
R119	HO_2_ + CH_3_ < = > OH + CH_3_O
R155	CH_3_ + O_2_ < = > O + CH_3_O
R156	CH_3_ + O_2_ < = > OH + CH_2_O
R157	CH_3_ + H_2_O_2_ < = > HO_2_ + CH_4_
R158	2CH_3_(+ M) < = > C_2_H_6_(+ M)
R161	CH_3_ + CH_2_O < = > HCO + CH_4_
R170	CH_3_O + O_2_ < = > HO_2_ + CH_2_O

### Simulation condition

To reveal the effect of carbon dioxide on the kinetic characteristics of the methane explosion over wide methane equivalent ratios, the explosion of different methane concentrations within the explosion limit was simulated by using a higher initial temperature instead of the high-temperature heat source (> 650℃)^26^. In the present study, the methane explosion is simulated with the constant volume combustion bomb model, with the initial temperature of 1300 K, the initial pressure of 1 atm, and the reaction time of 0.02 s. The specific working conditions are presented in Table 2.

**Table 2:** Initial working conditions of methane explosion.

7 vol% CH_4_ (*φ* = 0.72)			9.5 vol% CH_4_(*φ* = 1)			11 vol% CH_4_(*φ* = 1.18)		
CO_2_	O_2_	N_2_	CO_2_	O_2_	N_2_	CO_2_	O_2_	N_2_
0	19.53	73.47	0	19.005	71.495	0	18.69	70.31
2	19.11	71.89	2	18.585	69.915	2	18.27	68.73
4	18.69	70.31	4	18.165	68.335	4	17.85	67.15
6	18.27	68.73	6	17.745	66.755	6	17.43	65.57
8	17.85	67.15	8	17.325	65.175	8	17.01	63.99
10	17.43	65.57	10	16.905	63.595	10	16.59	62.41

## Calculation results and analysis

### Pressure and temperature

**Figure 1:**
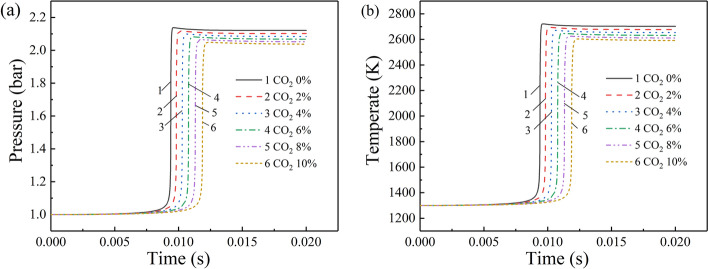
Variation of explosion pressure and temperature with time under different CO_2_ volume fractions at 7% CH_4_: (**a**) explosion pressure and (**b**) explosion temperature.

The variations of the pressure and temperature during the explosion process of 7% CH_4_–air with different CO_2_ additions are plotted in Fig. 1. With the increase of the CO_2_ volume fraction, the explosion time prolongs and the explosion pressure and temperature decrease monotonously. When the volume fraction of CO_2_ increases from 0 to 10%, The maximum gas explosion pressure decreases from 2.12 to 2.04 MPa with the decrease rates of 3.77%. The maximum temperature decreases from 2702.882 K to2591 K with the decline rates of 4.14%. These results indicate that the increase of the volume fraction of CO_2_ would suppress the gas explosions. This conclusion agrees with the effect of water addition on methane explosion^27^.

**Figure 2:**
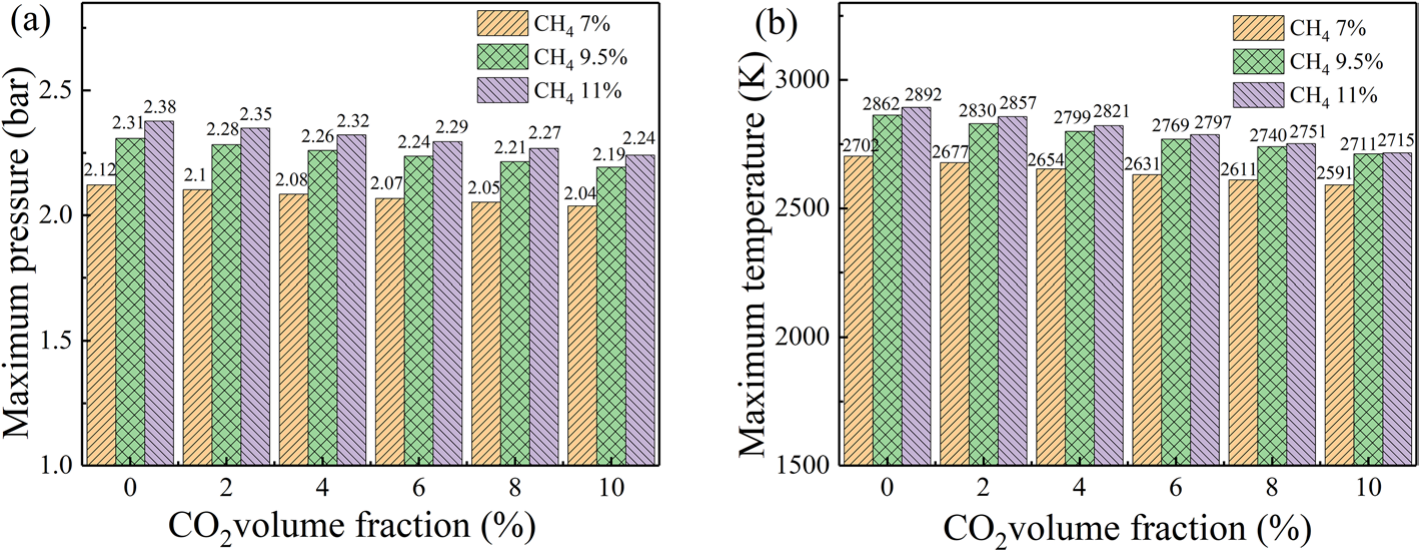
Variation of explosion pressure and temperature with CO_2_ volume fraction under different methane volume fractions: (**a**) explosion pressure and (**b**) explosion temperature.

Figure 2 further displays the influence of the CO_2_ volume fraction on the maximum explosion pressure and explosion temperature with different methane volume faction. As seen, the maximum explosion pressure and explosion temperature decrease with the increase of the CO_2_ volume fraction under all the methane volume fraction. The larger the methane volume fraction, the greater the maximum explosion pressure decrease, and the better the suppression effect on the methane explosion. When the volume fraction of methane is 7%, 9.5%, 11%, the maximum explosion pressure of adding 10% CO_2_ is reduced by 3.9% compared with the case with no addition in Fig. 2a. As Fig. 2b shows, for methane with a volume fraction of 11%, the explosion temperature is more sensitive to changes in the CO_2_ volume fraction than for 7% and 9.5% volume fractions. When the volume fraction of methane is 7%, 9.5%, 11%, the explosion temperature of the addition of 10% CO_2_ decreases by 4.2%, 5.3%, 6.2% compared with the case with no addition. The results indicate that the inhibitory effect of CO_2_ addition on the methane explosions increases as the increase of the methane concentration.

### Free radicals

**Figure 3:**
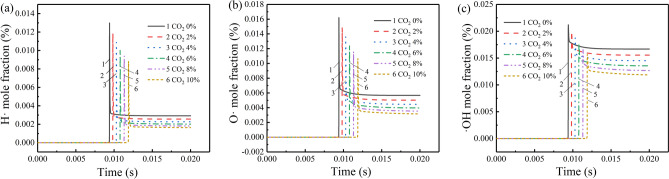
Variation of free radicals concentration with time under different CO_2_ concentrations at 7% CH_4_: (**a**) free radical H, (**b**) free radical O, and (**c**) free radical OH.

The essence of gas explosion is a complex thermal chain reaction. The chain-branching and chain-propagating reactions initiated by free radicals play an important role in the chemical reaction. H + O_2_ <  =  > O + OH and H + CH_4_ <  =  > CH_3_ + H_2_, which are the most dominant chain branching reactions of methane explosion^28^, contribute to the product amounts of free radicals O and OH^29^. When the mixed gas absorbs enough energy, the molecular chain breaks. Then, the number of free radicals H, O and OH begin to soar to form a chemical reaction active center with a high concentration of free radicals, which eventually leads to the explosion. As shown in Fig. 3, when the volume fraction of methane is 7% with no CO_2_ addition, the maximum mole fraction of the free radicals H, O, and OH are 0.013, 0.016, and 0.021, respectively. Because the addition of CO_2_ increases the probability of free radicals collision with the third body to form low-activity stable molecules, as the increase of the CO_2_ volume fraction, the location of peak concentration of free radicals prolongs and the peak concentrations of the free radicals H, O, and OH decrease.

**Figure 4:**
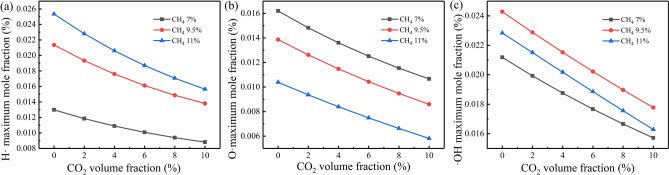
Variation of maximum mole fraction of free radicals with CO_2_ volume fraction under different methane volume fractions: (**a**) free radical H, (**b**) free radical O, and (**c**) free radical OH.

Figure 4 shows the effect of CO_2_ addition on the peak concentration of radical H, O, and OH over φ = 0.72,1,1.18. It can be found that the CO_2_ addition reduces the peak concentration of all the investigated radicals. The greater the methane volume fraction, the greater the decrease rate of radicals H and OH, and the smaller the decrease rate of radical O. When the volume fraction of CO_2_ is constant, the increase of the volume fraction of methane leads to the increase of the maximum mole fraction of radical H· and the decrease of the maximum mole fraction of radical O. For radical OH, its maximum mole fraction first increases and then decreases with the location of peak value of 9.5%. The larger the equivalence ratio of CH_4_, the less O_2_ in the mixture, which increases the number of CH_4_ molecules and decreases the number of O_2_ molecules in the unit volume of the reactant. The concentration of radical H increases, and the concentration of radical O decreases. At the same time, with the increase of CH_4_ concentration, the elementary reaction step R52: H + CH_3_(+ M) <  =  > CH_4_(+ M), R11: O + CH_4_ <  =  > OH + CH_3_ tend to promote the consumption of CH_4_. It also explains the appearance of Fig. 2.

### Gas products

**Figure 5:**
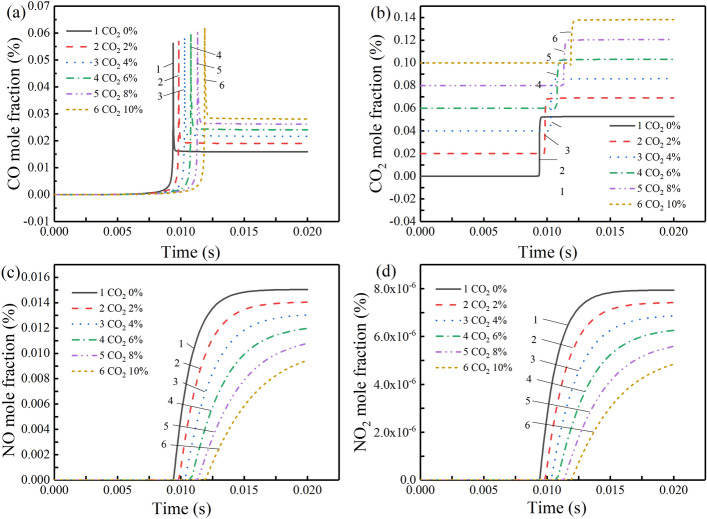
Variation of product concentration of some gases with time under different CO_2_ volume fractions at 7% CH_4_: (**a**) CO, (**b**) CO_2_, (**c**) NO, and (**d**) NO_2_.

The catastrophic gases, such as CO, CO_2_, NO, NO_2_, produced in the gas explosions process are the major cause of casualties^30^. After adding CO_2_, the change of the mole fraction of catastrophic gas with 7% CH_4_–air is shown in Fig. 5.

As seen, with the increase of the CO_2_ volume fraction, the mole fraction of CO is increased, whereas the mole fractions of CO_2_, NO, and NO_2_ are decreased. This is caused by elementary reaction R31: O_2_ + CO <  =  > O + CO_2_, R99: OH + CO <  =  > H + CO_2_, R120: HO_2_ + CO <  =  > OH + CO_2_, R132: CH + CO_2_ <  =  > HCO + CO, R153: CH_2_(S) + CO_2_ <  =  > CO + CH_2_O. When CO_2_ is added to the gas mixture, the initial concentration of CO_2_ in the gas mixture increases, which causes the above reaction is easier to happen toward to the direction of CO_2_ consumption, which results in a large amount of CO. Figure 5a reveals that the mole fraction of CO reaches its peak first, then it reacts with the excess oxygen to form CO_2_, and eventually tends to a stable value. Under working condition 1, after gas explosion, the mole fractions of CO, CO_2_, NO, and NO_2_ are 0.0159, 0.0527, 0.0150, and 7.94 × 10^−6^, respectively. Under working condition 6, after gas explosion, the mole fractions of CO, CO_2_, NO, and NO_2_ are 0.0281, 0.0382, 0.0094, and 4.84 × 10^−6^. the increase rate of CO is 76%, and the decrease rates of CO_2_, NO, and NO_2_ are 27%, 37%, and 39%.

Table 3 lists the effect of CO_2_ addition on the concentration of the catastrophic gas under different methane volume fractions. It shows that, φ = 0.72, 1, 1.18, with the increase of the CO_2_ volume fraction, the mole fraction of CO is increased, and the mole fractions of CO_2_, NO, and NO_2_ are decreased accordingly in all the investigated conditions. When the volume fraction of CO_2_ is 10%, with the increase in methane volume fraction, the volume fraction of CO rises while those of CO_2_, NO and NO_2_ fall. The above results indicate that the addition of CO_2_ plays a positive role in inhibiting the formation of NO and NO_2_ but promoting the formation of CO.

**Table 3:** Mole fractions of gas products under different working conditions.

Working condition	CO	CO_2_	NO	NO_2_
7% CH_4_	0.01590066	0.05270834	0.01503820	0.00000793779
7% CH_4_ + 10% CO_2_	0.02811000	0.03818010	0.00944985	0.00000484152
9.50% CH_4_	0.04258436	0.04812039	0.01105626	0.00000329051
9.5% CH_4_ + 10% CO_2_	0.06740572	0.01813410	0.00693919	0.00000190858
11% CH_4_	0.06146430	0.04155837	0.00759739	0.00000149968
11% CH_4_ + 10% CO_2_	0.09511225	0.00064950	0.00415820	0.00000070496

### Key reactions

**Figure 6:**
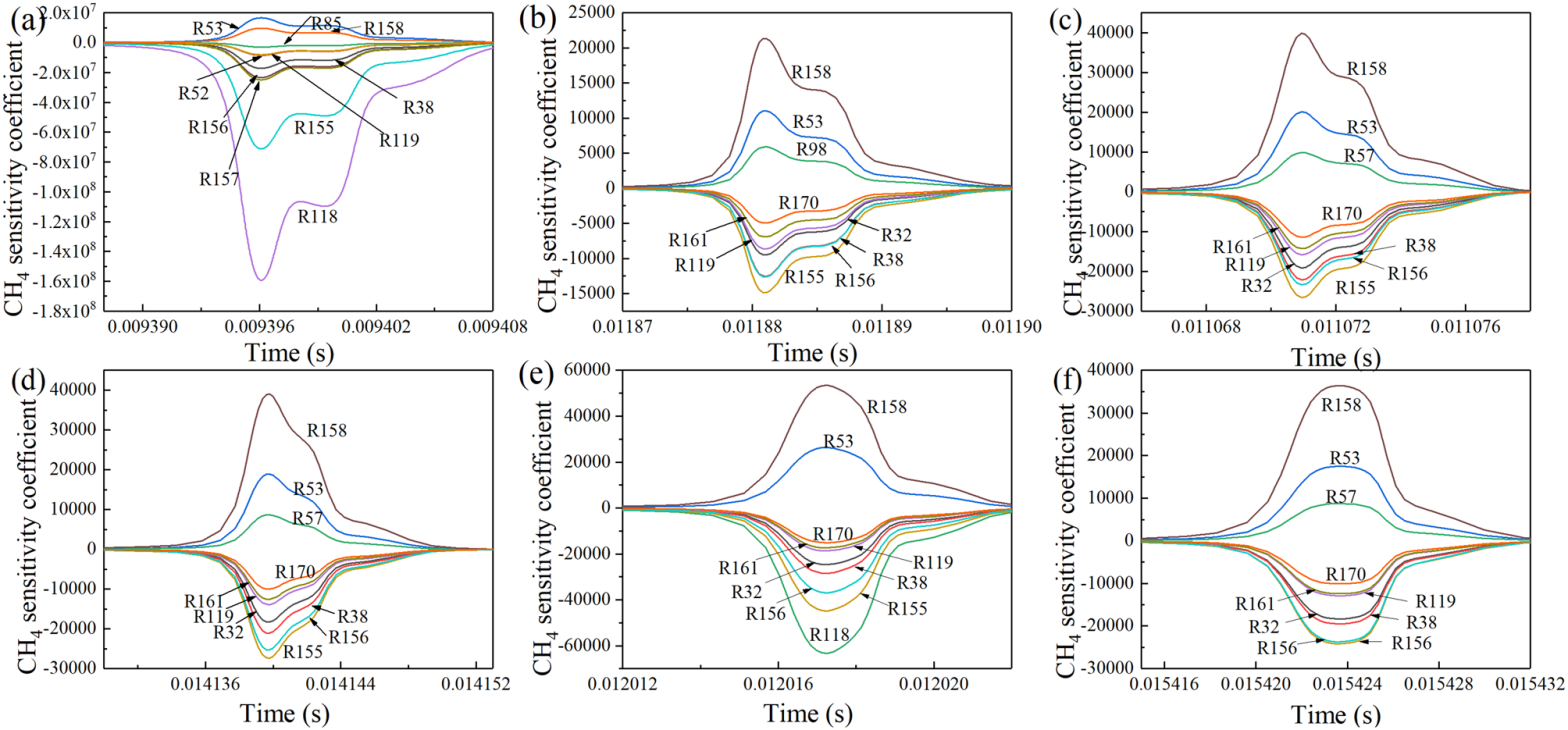
Key reaction steps affecting the change of CH_4_ mole fraction: (**a**) 7%CH_4_ 0%CO_2_, (**b**) 7%CH_4_ 10%CO_2_, (**c**) 9.5%CH_4_ 0%CO_2_, (**d**) 9.5%CH_4_ 10%CO_2_, (**e**) 11%CH_4_ 0%CO_2_, and (**f**) 11%CH_4_ 10%CO_2_.

The key elementary reaction steps during the methane explosion under different conditions are shown in Fig. 6. According to Fig. 6a, when 7% CH_4_-Air explodes, the key reaction steps inhibiting CH_4_ consumption are R53 and R158. Both reactions consume the free radicals H, O, and OH, which interrupt the chain reaction. The key reaction steps promoting CH_4_ consumption are R118, R155, R157, R156, R38, R52, R119, and R85. These reactions promote the formation of free radicals, and enhance the chain reaction.

According to Fig. 6b, after the addition of 10% CO_2_, the key elementary reaction steps inhibiting CH_4_ consumption change from R53 and R158 to R158, R53, and R98. The key reaction step promoting CH_4_ consumption change from R118, R155, R157, R156, R38, R52, R119, and R85 to R155, R156, R38, R32, R119, R161, and R170. The sensitivity coefficients of each elementary reaction step are decreased, and the time of the maximum sensitivity coefficient of each elementary reaction step prolongs; at the same time, the reduction amplitude of the coefficient to promote methane consumption is greater than to promote methane formation. This indicates that the change in methane concentration is affected by these reaction steps, the influence becomes weaker, and the addition of CO_2_ inhibits the combustion of methane.

**Figure 7:**
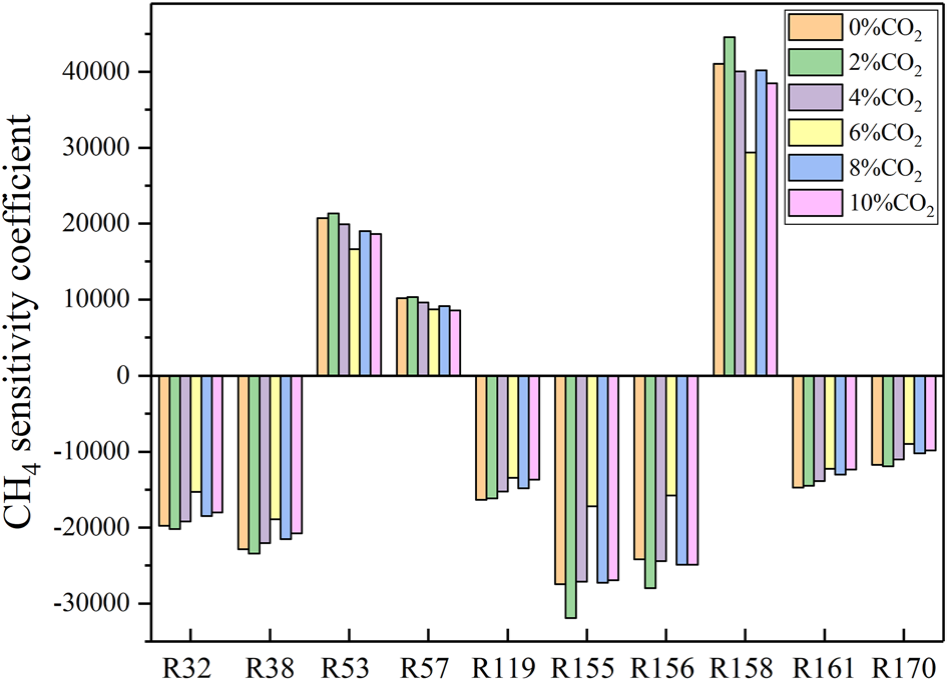
Effects of CO_2_ addition on the sensitivity coefficients of CH_4_ mole fraction. (9.5%CH_4_).

Figure 6c, d show that, when 9.5% CH_4_-Air explodes, the key elementary reaction steps inhibiting CH_4_ consumption are R158, R53, and R57, and the key elementary reaction steps promoting CH_4_ consumption are R155, R156, R38, R32, R119, R161, and R170. When 10% CO_2_ was added, the key elementary reaction steps promoting and inhibiting CH_4_ consumption do not change. The effects of CO_2_ addition on the sensitivity coefficients of CH_4_ mole fraction under the methane volume fraction of 9.5% are given in Fig. 7. It can be seen that the sensitivity coefficients of these elementary reactions drop gradually with the increase of CO_2_ concentration. Meanwhile, the time when the sensitivity coefficient of each elementary reaction step reaches the maximum value moves back. This means that for the methane explosion with a methane equivalence ratio of 1, the addition of CO_2_ has little effect on the change in the methane concentration during the explosion, but inhibits the methane explosion.

As Fig. 6e, f show, when 11% CH_4_-Air explodes, the key elementary reaction steps inhibiting CH_4_ consumption are R158 and R53, and the key elementary reaction steps promoting CH_4_ combustion are R118, R155, R156, R38, R32, R119, R161, and R170. When 10% CO_2_ was added, the key elementary reaction steps inhibiting CH_4_ consumption are R158, R53, and R57, and the key elementary reaction steps promoting CH_4_ combustion are R155, R156, R38, R32, R119, R161, and R170. The key elementary reaction steps promoting and inhibiting CH_4_ consumption are basically the same as those without CO_2_, but the sensitivity coefficients of each elementary reaction step are decreased, and the reduction amplitude of the coefficient of promoting CH_4_ consumption is greater than inhibiting CH_4_ consumption. This indicates that the addition of CO_2_ inhibits the process of methane explosion to a certain extent.

## Conclusion

In this study, 2%, 4%, 6%, 8%, and 10% CO_2_ were sequentially filled into a mixed gas with different methane concentrations. The explosion reaction time prolonged as the increase of CO_2_ volume fraction and the maximum pressure and temperature of the methane explosion were significantly reduced compared with the case with no CO_2_ addition. If the volume fraction of CO_2_ is constant, with the increase of methane concentration, the inhibitory effect of CO_2_ on methane explosion was increasingly effective.

In the fuel-lean, stoichiometric and fuel-rich conditions, the peak mole fraction of free radicals decreased with the increase of the CO_2_ volume fraction. When the volume fraction of CO_2_ is constant, as the volume fraction of methane increased, the maximum mole fraction of radical H increased, while the maximum mole fraction of radical O decreased. For radical OH, its maximum mole fraction first increased and then decreased with the location of peak value of 9.5%.

After 10% CO_2_ was added to the 7% CH_4_-Air, the mole fraction of CO increased by 76%, while the mole fractions of CO_2_, NO, and NO_2_ decreased by 27%, 37%, and 39%, respectively. The higher the volume fraction of CH_4_, the more CO was produced after the addition of CO_2_. Although the addition of CO_2_ played a positive role in inhibiting the formation of NO and NO_2_, it promoted the formation of CO.

The addition of CO_2_ changed the key elementary reaction steps affecting CH_4_ concentration, and the time of the maximum sensitivity coefficient of each reaction step prolonged. When CH_4_ was in a fuel-lean, stoichiometric and fuel-rich conditions, the sensitivity coefficient of each key elementary reaction step was reduced, and the reduction amplitude of the coefficient promoting methane consumption was larger than inhibiting the consumption, indicated that the addition of CO_2_ could inhibit CH_4_ explosion.

In general, the methane explosion can be inhibited by adding CO_2_, and the greater the volume fraction of CO_2_, the better the inhibition effect. However, more CO will be produced under a higher methane concentration. In the application of CO_2_ addition to gas explosion suppression, it is necessary to consider the possibility of CO poisoning under practical working conditions.
